# Preoperative sequential short-course radiation therapy and FOLFOX chemotherapy versus long-course chemoradiotherapy for locally advanced rectal cancer: a multicenter, randomized controlled trial (SOLAR trial)

**DOI:** 10.1186/s12885-023-11363-7

**Published:** 2023-11-03

**Authors:** Min Kyu Kang, Soo Yeun Park, Jun Seok Park, Hye Jin Kim, Jong Gwang Kim, Byung Woog Kang, Jin Ho Baek, Seung Hyun Cho, An Na Seo, Duck-Woo Kim, Jin Kim, Se Jin Baek, Ji Hoon Kim, Ji Yeon Kim, Gi Won Ha, Eun Jung Park, In Ja Park, Chang Hyun Kim, Hyun Kang, Gyu-Seog Choi

**Affiliations:** 1 Department of Radiation Oncology, Kyungpook National University Chilgok Hospital, School of Medicine, Kyungpook National University, Daegu, South Korea; 2 Colorectal Cancer Center, Kyungpook National University Chilgok Hospital, School of Medicine, Kyungpook National University, 807 Hogukro, Buk-gu, Daegu, 41404 South Korea; 3 Department of Oncology/Hematology, Kyungpook National University Chilgok Hospital, School of Medicine, Kyungpook National University, Daegu, South Korea; 4 Department of Radiology, Kyungpook National University Chilgok Hospital, School of Medicine, Kyungpook National University, Daegu, South Korea; 5 Department of Pathology, Kyungpook National University Chilgok Hospital, School of Medicine, Kyungpook National University, Daegu, South Korea; 6 Department of Surgery, Seoul National University Bundang Hospital, Seongnam, South Korea; 7 Division of Colon and Rectal Surgery, Department of Surgery, Korea University College of Medicine, Seoul, South Korea; 8 Division of Colorectal Surgery, Department of Surgery, Incheon St. Mary’s Hospital, the Catholic University of Korea, Incheon, South Korea; 9 Division of Colorectal Surgery, Department of Surgery, Chungnam National University Hospital, Daejeon, South Korea; 10 Research Institute of Clinical Medicine of Jeonbuk National University-Biomedical Research Institute of Jeonbuk National University Hospital, Jeonju, Jeonbuk, South Korea; 11 Division of Colon and Rectal Surgery, Department of Surgery, Gangnam Severance Hospital, Yonsei University College of Medicine, Seoul, South Korea; 12 Division of Colon and Rectal Surgery, Department of surgery, , University of Ulsan College of Medicine and Asan Medical Center, Seoul, South Korea; 13 Department of Surgery, Chonnam National University Hwasun Hospital and Medical School, Hwasun, South Korea; 14 Department of Anesthesiology and Pain Medicine, Chung-Ang University College of Medicine, Seoul, South Korea

**Keywords:** Rectal neoplasm, Neoadjuvant radiotherapy, Short-course radiation, Consolidation chemotherapy, Disease-free survival, Randomized controlled phase II trial, Protocol

## Abstract

**Background:**

Preoperative (chemo)radiotherapy has been widely used as an effective treatment for locally advanced rectal cancer (LARC), leading to a significant reduction in pelvic recurrence rates. Because early administration of intensive chemotherapy for LARC has more advantages than adjuvant chemotherapy, total neoadjuvant therapy (TNT) has been introduced and evaluated to determine whether it can improve tumor response or treatment outcomes. This study aims to investigate whether short-course radiotherapy (SCRT) followed by intensive chemotherapy improves oncologic outcomes compared with traditional preoperative long-course chemoradiotherapy (CRT).

**Methods:**

A multicenter randomized phase II trial involving 364 patients with LARC (cT3–4, cN+, or presence of extramural vascular invasion) will be conducted. Patients will be randomly assigned to the experimental or control arm at a ratio of 1:1. Participants in the experimental arm will receive SCRT (25 Gy in 5 fractions, daily) followed by four cycles of FOLFOX (oxaliplatin, 5-fluorouracil, and folinic acid) as a neoadjuvant treatment, and those in the control arm will receive conventional radiotherapy (45–50.4 Gy in 25–28 fractions, 5 times a week) concurrently with capecitabine or 5-fluorouracil. As a mandatory surgical procedure, total mesorectal excision will be performed 2–5 weeks from the last cycle of chemotherapy in the experimental arm and 6–8 weeks after the last day of radiotherapy in the control arm. The primary endpoint is 3-year disease-free survival, and the secondary endpoints are tumor response, overall survival, toxicities, quality of life, and cost-effectiveness.

**Discussion:**

This is the first Korean randomized controlled study comparing SCRT-based TNT with traditional preoperative LC-CRT for LARC. The involvement of experienced colorectal surgeons ensures high-quality surgical resection. SCRT followed by FOLFOX chemotherapy is expected to improve disease-free survival compared with CRT, with potential advantages in tumor response, quality of life, and cost-effectiveness.

**Trial registration:**

This trial is registered at Clinical Research Information under the identifier Service KCT0004874 on April 02, 2020, and at Clinicaltrial.gov under the identifier NCT05673772 on January 06, 2023.

**Supplementary Information:**

The online version contains supplementary material available at 10.1186/s12885-023-11363-7.

## Background

Preoperative chemoradiotherapy (CRT) has long been the established treatment for locally advanced rectal cancer (LARC), effectively reducing the risk of local recurrence [1, 2]. However, despite the benefits, approximately one-third of patients with LARC experience relapse following preoperative CRT and surgery, with distant metastasis being the most common pattern of failure [2–6]. Recently, promising results of total neoadjuvant therapy (TNT) have been achieved in two trials: reduced disease-related treatment failure in the RAPIDO trial [4, 6] and increased disease-free survival (DFS) in the PRODIGE 23 trial [6]. As a results, TNT has emerged as the recommended treatment for LARC, emphasizing the need for timely and effective systemic therapy to eradicate micrometastases [7].

The main distinction between TNT and traditional preoperative CRT lies in the use of intensive chemotherapy before surgery. The RAPIDO and PRODIGE 23 trials differ in terms of tumor location, cT stage, cN stage, and circumferential resection margin status. Moreover, the TNT protocols vary in the sequence of treatments, types and cycles of neoadjuvant chemotherapy, and types of radiotherapy employed [4–6]. Currently, ongoing trials are evaluating different neoadjuvant treatment options for LARC.

While preoperative CRT has served as the standard treatment for LARC over the years and continues to be utilized in many countries, including South Korea [8, 9], short-course radiotherapy (SCRT) followed by delayed surgery has demonstrated comparable down-staging and surgical outcomes to CRT [10, 11]. Although systemic chemotherapy can be combined with both types of radiotherapy, a greater number of cycles of systemic chemotherapy can be scheduled with SCRT than CRT during the same treatment period.

Herein, we propose a neoadjuvant treatment protocol consisting of SCRT and four cycles of FOLFOX chemotherapy. Our randomized controlled trial aims to compare the 3-year DFS of patients with LARC treated with either preoperative CRT or SCRT followed by FOLFOX chemotherapy before total mesorectal excision (TME). We hypothesized that our protocol would yield superior systemic treatment effects compared to traditional CRT, reducing the total treatment period from the initiation of neoadjuvant treatment to the completion of adjuvant chemotherapy, and enhancing patient convenience.

## Methods

### Study setting

**Fig. 1:**
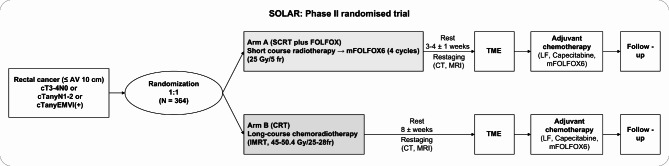
Flowchart of the SOLAR trial. SCRT, short-course radiotherapy; FOLFOX, 5-fluorouracil with oxaliplatin; CRT, chemoradiotherapy; CT, computed tomography; MRI, magnetic resonance imaging; TME, total mesorectal excision

The present study is a parallel-group, multicenter, superiority, randomized, phase II trial. The participants will be enrolled in nine tertiary academic hospitals in South Korea. The trial flow scheme is presented in Fig. 1.

### Endpoints

The primary endpoint is the 3-year DFS, which is measured from the time of randomization until locoregional failure, metastasis, secondary cancer, or all-cause mortality. The data of patients who have not experienced any events by the time of the analysis will be censored at the last follow-up date. The Kaplan-Meier method will be used to calculate the survival rates. The secondary endpoints are the pathological complete response (pCR) rate; proportions of pathologic tumor regression grade (TRG) (Dworak [12], Mandard [13], AJCC TRG system [14]; R0 resection (microscopically clear resection) rate, 60-day postoperative surgical morbidity and mortality; treatment-related toxicity using the Common Terminology Criteria for Adverse Events version 5.0 (CTCAE v5.0); compliance with the study protocol defined as the completion of preoperative treatment, surgery, and adjuvant treatment; TRG based on magnetic resonance imaging (MRI) [15]); quality of TME [16]; 5-year DFS; 3/5-year overall survival (OS); loco-regional recurrence; distant metastasis; 3-year peripheral neuropathy grade (CTCAE v5.0); questionnaires of quality of life (QoL) using the Korean version of the European Organization for the Research and Treatment of Cancer Quality of Life Questionnaire Core 30 (EORTC QLQ-C30) version 3.0 [17]; QLQ-CR29 [18]; Low Anterior Resection Syndrome (LARS) score [19]; 5-item version of the International Index of Erectile Function (IIEF-5) [20]; retrograde ejaculation from inclusion to 2 years after surgery; and cost-effectiveness (direct cost, indirect cost, and quality-adjusted life-year).

### Study population, screening, and randomization

Patients with LARC are eligible for enrollment in this trial. They will be identified by the investigators and referred to the institutional multidisciplinary team for eligibility verification. The primary investigator will take charge of the final enrollment based on the inclusion/exclusion criteria (Table 1). Eligible patients who are willing to participate receive details of the study. After providing written informed consent at each hospital, the participants will be randomly assigned to either Arm A (experimental arm: SCRT plus mFOLFOX6) or Arm B (control arm: CRT). Block randomization at a ratio of 1:1 will be performed and managed by the central clinical research coordinator.

**Table 1:** Inclusion and exclusion criteria

Inclusion criteria	Exclusion criteria
1) A male/female adult aged between 20 and 75 years 2) Race: Asian 3) Eastern Cooperative Oncology Group performance status 0–2 4) Pathologically confirmed rectal cancer (rectal cancer located 10 cm or low from the anal verge in pelvis MRI) 5) Histologic type: adenocarcinoma, mucinous carcinoma, and signet ring cell carcinoma 6) Locally advanced rectal cancer with one or more of the following factors based on pelvis MRI: cTanyN1-2, cT3-4N0, or presence of extramural vascular invasion 7) MRI findings such as pelvic lymph node metastasis, anal sphincter invasion, and T4b are not included in the exclusion criteria, which cases will be enrolled by the researcher’s discretion 8) Patients with appropriate organ (bone marrow, kidney, liver) function 9) A person who understands the study and willing to provide informed consent	1) Colon cancer or rectal cancer located more than 10 cm from the anal verge 2) Stage I rectal cancer (clinical stage cT1-2N0) 3) Clinically or pathologically diagnosed distant metastasis (cTanyNanyM1) 4) Familial adenomatous polyposis 5) Hereditary nonpolyposis colorectal cancer 6) History of chemotherapy or radiotherapy within 6 months 7) History of colorectal cancer or other malignant tumors within 5 years 8) Currently under treatment of malignant tumors (except for cured nonmelanoma skin or in situ cervical cancer) 9) Comorbidities that make it difficult to undergo chemotherapy or radiotherapy 10) Bone marrow suppression with neutrophil count < 2 × 10^9^/L or platelet count < 100 × 10^9^/L prior to the first chemotherapy 11) Peripheral sensory neuropathy with functional impairment (grade 2 or higher) 12) Severe renal function impairment (creatinine cleaning rate 30 ml/min or less) 13) Severe hepatic dysfunction 14) Genetic problems such as galactose intolerance, Lapp lactase deficiency, or glucose-galactose malabsorption 15) Taking tegafur, gimeracil, oteracil complex and those within 7 days of discontinuation 16) Taking sorivudine, brivudin 17) Significant heart disease or myocardial infarction within the last 6 months 18) Hereditary diseases or history of coagulopathy 19) Central nervous system disorders with disability or mental disorders 20) Pregnant or lactating women 21) Currently participating in other clinical trials or receiving research medication 22) Unhealed wounds, fractures, peptic ulcers, abscesses in the abdominal cavity 23) Active gastrointestinal bleeding 24) Active infection requiring injection with antibiotics 25) Emergency surgery 26) History of hypersensitivity to drug in the study protocol 27) Dihydropyrimidine dehydrogenase deficiency 28) Not willing to participate

MRI, magnetic resonance imaging

### Preoperative treatment

The participants in Arm A will receive SCRT (25 Gy in 5 fractions on consecutive days) followed by four cycles of mFOLFOX6 at 2-week intervals. The mFOLFOX6 regimen is composed of leucosodium 400 mg/m^2^ (or levoleucovorin 200 mg/m^2^ or leucovorin 400 mg/m^2^) and oxaliplatin (85 mg/m^2^) administered IV for 2-h and 5-fluorouracil (5-FU) 400 mg/m^2^ bolus followed by continuous infusion of 5-FU 2400 mg/m^2^ for over 46 h. The participants assigned to Arm B will receive CRT: a total dose of 45–50.4 Gy in 25–28 fractions given 5 times a week concurrently with capecitabine at a dose of 825 mg/m^2^ (twice daily) or bolus 5-FU 400 mg/m^2^ with leucovorin 20 mg/m^2^ (or levoleucovorin 10 mg/m^2^) on 1–4 days of the first and fifth week of CRT. Radiotherapy will be administered using 3-dimensional conformal or intensity-modulated radiotherapy. Clinical target volume will be delineated according to the guideline of the Radiation Therapy Oncology Group [21]. Details of radiation treatment planning will be determined at the discretion of participating radiation oncologists. Treatment-related toxicity will be monitored throughout the preoperative phase and chemotherapy dose modification can be made according to the protocol and investigator’s decision.

### Surgery

The surgery timing from the start of radiotherapy is comparable between the two arms. After the completion of preoperative treatments, the tumor will be reevaluated with computed tomography (CT) scan and MRI within 4 weeks before the scheduled surgery. TME will be performed at 2–5 weeks from the last cycle of preoperative chemotherapy in Arm A and at 6–8 weeks (maximum resting period of 10 weeks) after the last day of radiotherapy in Arm B. Surgical resection aims at achieving complete resection of primary tumor using TME with regional lymph node dissection. The type of surgical approach (open, laparoscopic, or robotic), starting point and direction of mesocolic and mesorectal detachment, location of major vascular ligation, splenic flexure mobilization, intestinal anastomosis, stoma creation, and surgical instruments will follow each investigator’s discretion. Postoperative complications will be recorded on the case report form for up to 60 days following surgery.

Surgical resection will be performed by a colorectal surgeon with sufficient knowledge and experience in rectal cancer surgery and treatment. All participating surgeons should have completed a fellowship in colorectal surgery and experience in more than 50 cases of rectal cancer surgery. The designated questionnaire presented in Supplementary Table 1 will be used for the recruitment of hospitals and surgeons. Each hospital has designated radiation oncologist(s), oncologist(s), pathologist(s), and radiologist(s) specializing in rectal cancer. The historical data on TME performed by the participating hospitals and surgeons is presented in Supplementary Fig. 1. The surgeons must submit unedited rectal cancer surgery videos to obtain approval from the research steering committee before the study participation. To ensure the quality of surgery and standardized surgical procedures, discussion on rectal cancer surgery and video presentation will be performed at the investigator meetings twice a year during the enrollment period.

### Postoperative chemotherapy

Adjuvant chemotherapy is recommended for all patients and initiated within 4 weeks following TME. The decision to use adjuvant chemotherapy and the timing of its initiation will be determined by the investigators in charge of chemotherapy based on each patient’s postoperative condition. The duration of adjuvant chemotherapy is about 4 months, resulting in a total chemotherapy period of 6 months. Both arms will receive adjuvant chemotherapy according to the pathological stage as follows: capecitabine or 5-FU with leucovorin (or leucosodium or levoleucovorin) for ypStages 0 to I and mFOLFOX 6 for ypStages II to III. In case of Stage IV confirmed before or at the time of surgery, additional treatment is determined by the institutional multidisciplinary team.

### Pathological evaluation

Pathological evaluation will be performed by the dedicated pathologists specializing in rectal cancer at each institution according to the AJCC Cancer Staging manual, 8th edition [14], and recorded using the pathological report form of the study, which includes the number of harvested/metastatic lymph nodes, length of proximal/distal/circumferential resection margin, and TRG using at least one of the three TRG systems (AJCC [14], Dworak [12], and Mandard [13] TRG system). The quality of the resected specimen will be classified for mesorectum and sphincter complex in case of abdominoperineal resection [22].

### Follow-up

Each participant will undergo regular follow-up until 5 years following surgery for history and morbidity/toxicity assessment, physical examination, measurement of carcinoembryonic antigen (CEA), and assessment of questionnaires of QoL. Toxicity will be evaluated and recorded according to CTCAE v5.0. Furthermore, CEA will be measured at 3 months following surgery and every 6 months thereafter. Abdominopelvic and chest CT scans will be performed every 6 months during the first 2 years and then annually during the following 3 years. QoL assessment using EORTC questionnaires QLQ-C30 and QLQ-CR29, LARS score for all patients, and IIEF-5 for male patients will be performed at 3, 6, 12, and 24 months.

### Translational research

Translational research using tumor tissue and blood will be conducted to interpret the clinical outcomes, treatment response, and prognosis in association with the molecular biological characteristics of rectal cancer. A specified protocol for the collection and preparation of fresh tissue and blood at different treatment stages will be given to all participating hospitals. Participation in the translational components of this trial is optional and subject to regional capacities. Samples will be collected and used after obtaining participant consent.

### Sample size calculation

The 3-year DFS following preoperative CRT is expected to be 70% based on literature [23–27]. We hypothesized that the experimental arm would have a 10% improvement in 3-year DFS compared with the control group. This corresponds to a hazard ratio of 0.626 between the two arms (experimental /control). Assuming an 80% statistical power, a one-sided alpha level of 0.05, a minimal accrual duration of 36 months, and a minimal follow-up of 36 months, a total of 113 events will be needed to observe the expected difference using the one-sided log-rank test. Assuming a 5% rate of patients who are not informative or lost to follow-up, a total of 364 patients will be enrolled in this study, with 182 patients in each group.

### Statistical analysis

The primary endpoint will be analyzed according to the intention-to-treat principle in patients who are randomized and receive preoperative treatments. Secondary analyses will be conducted using the per-protocol principle, considering only patients with sufficient compliance with the protocol. The Kaplan–Meier curve will be used to analyze the survival outcomes and the log-rank test for the prognostic factors. The chi-squared test will be employed to compare categorical data between the two groups and Student’s *t*-test for continuous variables. The effect of covariates on the endpoints will be evaluated using the Cox proportional-hazards model. QoL, assessed using questionnaires, will be expressed as mean, standard deviation, median, minimum, and maximum values. A mixed model for repeated-measure analysis with the assumption of an unconstructed covariance structure will be applied for QoL comparison. Least-square mean estimates will be calculated for *post hoc* analyses to compare the two treatment groups at each time point and evaluate any changes from the baseline scores. All tests will be two-sided, and *P*-value < 0.05 will be considered to indicate statistically significant differences. This trial has no plan for interim analysis. Missing data for the primary endpoint will be censored at either the last assessment date or the trial deadline, whichever is earlier. Because the primary endpoint is presented as recurrence or not, there are no outlier data. If there are missing values for the secondary endpoints, the last observation carried forward method will be employed to impute for the missing data with the most recently available data, and the Grubb and Cochran tests will be adopted to determine outliers if suspected.

### Data collection and management

Data will be collected using an electronic clinical research form (eCRF) system approved by the steering committee. Each study site is responsible for data entry, and the principal investigator is responsible for confirming the final data. During the study period, a monitoring team from the contract research organization (CRO) will regularly contact and visit all sites, with occasional visits as necessary. To ensure that the study is conducted according to the protocol, the completeness, consistency, and accuracy of the data entered in the eCRF will be evaluated at each visit of the CRO. All adverse events are graded according to CTCAE v5.0 and recorded in the eCRF. The investigators are responsible for evaluating the causal relationship between protocol treatments and each event. Furthermore, they will report severe adverse events to both their institutional review board and the principal investigator. The independent Data and Safety Monitoring Board (DSMB) will monitor the recruitment, reported adverse events, and data quality when 10% (36), 30% (109), 50% (182), 70% (255), and 100% (364) of the participants are enrolled. The goal is to ensure that the study conforms to the current standards of Good Clinical Practice, with focus on the safety interests of the patients. The DSMB will provide the principal investigator with recommendations regarding potential trial modification, continuation, or premature termination.

### Current status

The study protocol was approved by the ethics committee of Kyungpook National University Chilgok Hospital on October 17, 2019 (No. KNUCH 2019-09-004-001) as well as the approval of ethics committees of all other participating institutions. Patients have been enrolled in the study since September 2021, and as of July 16, 2023, a total of 182 patients have been recruited.

## Discussion

Recent clinical studies have achieved promising results in reducing distant metastasis in LARC using TNT compared with surgery first or traditional preoperative CRT followed by surgery. Although the long-term outcomes of TNT in terms of OS and DFS are still awaited, TNT has emerged as an alternative to conventional treatment, potentially redefining the standard of care. At the time of launching the present trial, only one randomized clinical trial (POLISH II [27]) has been published. Thereafter, several randomized clinical trials using TNT have been published (RAPIDO [4], PRODIGE23 [6], STELLAR [5], FOWARC [28], CAO/ARO/AIO-12 [29], and OPRA [30]). These clinical trials differ in terms of the inclusion criteria, treatment schedules, type of radiotherapy and chemotherapy, and primary endpoints, the details of which can be found in Kang’s review article [31]. Because there are various combinations of them, a head-to-head comparison among all currently developed regimens is impossible.

This SOLAR trial compares the neoadjuvant treatment protocols (SCRT followed by four cycles of mFOLFOX6 vs. CRT) in rectal cancer with cT3–4, cN+, or extramural venous invasion. This trial is designed for mid- or low rectal cancer (tumor height  ≤ 10 cm from the anal verge) as upper rectal cancer can sometimes be treated like colon cancer and surgery can be modified as tumor-specific mesorectal excision [32]. As regards the chemotherapy duration, we adopted four cycles of mFOLFOX6 because the ypCR rate was as low as 11% with SCRT followed by three cycles of mFOLFOX6 in our initial experience [33]. Meta-analysis of 17 studies comparing SCRT followed by consolidation chemotherapy with CRT supports using at least four cycles of consolidation chemotherapy following SCRT to achieve improved ypCR and DFS over preoperative CRT alone [34]. Because SCRT followed by four cycles of CAPOX achieved significantly higher ypCR rate and 3-year OS than CRT alone in the STELLAR trial [5] involving patients with cT3–4 or cN+, the results of this SOLAR trial are awaited.

Although the RAPIDO trial used SCRT in a TNT arm, it compared a TNT regimen with longer chemotherapy duration with conventional CRT in LARC with high-risk features such as cT4, mesorectal fascia involvement, cN2, lateral lymph node metastasis, and extramural venous invasion [4]. The PRODIGE 23 trial included patients with LARC (cT3–4 or cN+) and administered induction chemotherapy with FOLFIRINOX in a TNT group [6]. The CAO/ARO/AIO-12 [29] and OPRA [30] trials compared induction and consolidation chemotherapies, and the results favored consolidation chemotherapy in the aspects of ypCR and organ preservation.

In this SOLAR trial, all patients are planned to undergo TME, without the option for nonoperative management, which is a trending approach for LARC. We plan to develop criteria for accurately diagnosing clinical complete response by leveraging the clinical and pathologic outcomes of this study, thereby informing future treatment protocols involving watchful waiting.

Our current treatment protocol presents a practical alternative, addressing concerns about potential over-treatment associated with TNT and the missed treatment opportunity of a watchful waiting strategy.

Studies comparing the cost-effectiveness of various preoperative treatment options in patients with LARC are scarce. The direct medical cost was lower in SCRT than in CRT [35]. Practically, patients should visit the hospital for 25–30 consecutive days for CRT, which influences indirect cost and QoL. This can pose a particular challenge in situations where cancer treatment is centered at designated hospitals or in countries with low health-care budgets. Two recent trials, the POLISH II [36] and ESCORT trial [9], compared the cost-effectiveness of two preoperative strategies, namely, SCRT plus chemotherapy and CRT in patients with LARC. These trials demonstrated that SCRT plus consolidation chemotherapy was more cost-effective than CRT for direct and/or indirect costs. To achieve patient-centered care, new treatments should improve not only the oncological outcomes but also the QoL for patients undergoing treatment. In this regard, the SOLAR trial will also include cost-effectiveness analysis as a secondary endpoint.

The quality of surgical resection is well known to be a crucial factor in local control and sphincter preservation following preoperative treatment and surgical resection [37]. In particular, surgical quality assessment is important in evaluating the effects of new preoperative treatments when TME is performed. Recent clinical trials have reported a wide range of complete TME, R0 resection, and sphincter preservation rates for LARC. The rates of complete TME were 81% in POLISH II and 86% in PRODIGE 23 trials; however, the rates were not reported in the RAPIDO and STELLAR. Furthermore, in POLISH II, PRODIGE 23, RAPIDO, and STELLAR trials, the R0 resection rates ranged from less than 80 to 90% and the rates of a permanent stoma during the initial TME ranged from 14 to 48%. These outcome variations may be partly attributed to differences in the inclusion criteria among the studies. Although TME with sufficient quality is currently accepted as a logical approach, the quality control of surgery may not be as standardized as that for preoperative treatment protocols. Thus, we paid particular attention to surgical quality control in our study to minimize surgical outcome variations. We are only inviting surgeons who had completed a fellowship in colorectal surgery and met the minimum criteria for experience in TME. The TME procedures of each surgeon are evaluated by the research steering committee through a review of unedited video. The details of surgical procedures such as TME and sphincter preservation will be regularly discussed in the investigator meetings to maintain consensus on surgical procedures until enrollment of the last case.

## Conclusions

The present trial is a multicenter prospective randomized controlled trial comparing SCRT followed by mFOLFOX6 and CRT as two preoperative treatments for LARC. Based on the similar effect on local control between SCRT and CRT as well as the potential systemic effect of neoadjuvant systemic chemotherapy, we hypothesized that preoperative treatment with SCRT and mFOLFOX6 would lead to longer DFS. Quality control measures are implemented to reduce surgery-related complications and achieve competent sphincter preservation, while ensuring adequate local tumor control.

### Electronic supplementary material

Below is the link to the electronic supplementary material.

Supplementary Material 1

Supplementary Material 2

## Data Availability

Data sharing is not applicable to this article as no datasets were generated or analysed during the current study.

## Abbreviations

5-FU: 5-Fluorouracil
CRT: Chemoradiotherapy
CEA: Carcinoembryonic antigen
CRO: Contract research organization
CT: Computed tomography
CTCAE: Common Terminology Criteria for Adverse Events
DFS: Disease-free survival
DSMB: Data and Safety Monitoring Board
eCRF: Electronic clinical research form
EOTRC QLQ: European Organization for Research and Treatment of Cancer Quality of Life Questionnaire
IIEF: International Index of Erectile Function
ITT: Intention-to-treat
LARC: Locally advanced rectal cancer
LARS: Low Anterior Resection Syndrome
MRI: Magnetic resonance imaging
OS: Overall survival
pCR: Pathological complete response
QoL: Quality of life
SCRT: Short-course radiotherapy
TME: Total mesorectal excision
TNT: Total neoadjuvant therapy
TRG: Tumor regression grade
