# Supplementary material for: Preoperative sequential short-course radiation therapy and FOLFOX chemotherapy versus long-course chemoradiotherapy for locally advanced rectal cancer: a multicenter, randomized controlled trial (SOLAR trial)
Source: BMC Cancer. 2023 Nov 3;23:1059. doi: 10.1186/s12885-023-11363-7 (PMC10623855; doi:10.1186/s12885-023-11363-7)
Supplement: Supplementary file 1 — Supplementary Material 1 [file 12885_2023_11363_MOESM1_ESM.docx]

**Supplementary Table S1**. Information of institutions and surgeons

| 1. Does your institution hold regular multidisciplinary team meeting or conferences (i.e., tumor board) for rectal cancer?  1) Yes  2) No  2. Is there a designated radiation oncologist(s) for rectal cancer at your institution?  1) Yes  2) No  3. Is there a designated oncologist(s) for rectal cancer at your institution?  1) Yes  2) No  4. Is there a designated pathologist(s) for rectal cancer at your institution?  1) Yes  2) No  5. Is there a designated radiologist(s) for rectal cancer at your institution?  1) Yes  2) No  6. Have you completed any specialized training or fellowship in colorectal surgery?  1) Yes  2) No  7. Are you a designated colorectal surgeon for rectal cancer at your institution?  1) Yes  2) No  8. How many TMEs for rectal cancer are performed annually at your institution?  ( ) cases  9. How many years of experience do you have in performing TME for rectal cancer before participating in the SOLAR trial?  ( ) years  10. How many TMEs for rectal do you perform annually?  ( ) cases  11. How many TMEs have you performed before participating the SOLAR trial?  ( ) cases |
| --- |

TME, total mesorectal excision
