# Supplementary material for: Preoperative sequential short-course radiation therapy and FOLFOX chemotherapy versus long-course chemoradiotherapy for locally advanced rectal cancer: a multicenter, randomized controlled trial (SOLAR trial)
Source: BMC Cancer. 2023 Nov 3;23:1059. doi: 10.1186/s12885-023-11363-7 (PMC10623855; doi:10.1186/s12885-023-11363-7)
Supplement: Supplementary file 2 — Supplementary Material 2 [file 12885_2023_11363_MOESM2_ESM.docx]

**Supplementary Figure S1**. Description of the participating institutions and surgeons. (A) Number of annual total mesorectal excision (TME) performed at participating hospitals. (B) Participating surgeons’ years of experience in performing TME. (C) Number of annual TME performed by participating surgeons. (D) Total number of TME performed by surgeons before participating in the SOLAR trial.
